# An EEG Experimental Study Evaluating the Performance of Texas Instruments ADS1299

**DOI:** 10.3390/s18113721

**Published:** 2018-11-01

**Authors:** Usman Rashid, Imran Khan Niazi, Nada Signal, Denise Taylor

**Affiliations:** 1 Health & Rehabilitation Research Institute, Auckland University of Technology, Auckland 0627, New Zealand; imran.niazi@aut.ac.nz (I.K.N.); nada.signal@aut.ac.nz (N.S.); denise.taylor@aut.ac.nz (D.T.); 2 Centre for Chiropractic Research, New Zealand College of Chiropractic, Auckland 1060, New Zealand; 3 SMI, Department of Health Science and Technology, Aalborg University, Aalborg 9220, Denmark

**Keywords:** ADS1299, OpenBCI Cyton V3-32, electroencephalography (EEG), movement-related cortical potential (MRCP), brain computer interface (BCI), NuAmps

## Abstract

Texas Instruments ADS1299 is an attractive choice for low cost electroencephalography (EEG) devices owing to its low power consumption and low input referred noise. To date, there have been no rigorous evaluations of its performance. In this EEG experimental study we evaluated the performance of the ADS1299 against a high quality laboratory-based system. Two self-paced lower limb motor tasks were performed by 22 healthy participants. Recorded power across delta, theta, alpha, and beta EEG bands, the power ratio across the motor tasks, pre-movement noise, and signal-to-noise ratio were obtained for evaluation. The amplitude and time of the negative peak in the movement-related cortical potentials (MRCPs) extracted from the EEG data were also obtained. Using linear mixed models, no statistically significant differences (*p* > 0.05) were found in any of these measures across the two systems. These findings were further supported by evaluation of cosine similarity, waveform differences, and topographic maps. There were statistically significant differences in MRCPs across the motor tasks in both systems. We conclude that the performance of the ADS1299 in combination with wet Ag/AgCl electrodes is analogous to that of a laboratory-based system in a low frequency (<40 Hz) EEG recording.

## 1. Introduction

Texas Instruments ADS1299 is a system on chip (SOC) specifically designed for biopotential applications including electroencephalography (EEG) and electrocardiography (ECG). It has attractive electrical characteristics such as low input referred noise (1 μV_pp_), low power consumption (5 mW/channel), test signals for impedance measurement, and an SPI^™^ compatible interface [1]. In recent years, it has attracted considerable attention from the biomedical research community, both for clinical research and the development of mobile brain computer interfaces (BCIs).

ADS1299 has been used in multiple studies to evaluate hardware and software for EEG. These include testing 3D printed electrodes [2], ultra high impedance active electrodes [3], finger-based dry electrodes [4], and artefact rejection algorithms [5,6]. It has also been used in EEG experimental research to study neurophysiology in both humans [7,8,9,10] and mice [11]. Importantly, these studies are making decisions about the relative merits of these technologies and experimental outcomes based on parameters of the EEG recorded with the ADS1299.

With an increased interest in mobile brain computer interfaces [12,13,14], engineers and researchers have sought front-ends which are low cost, have a small form factor, and possess good electrical characteristics [15]. Consequently, in the past two years alone, they have appeared in six novel BCIs, namely, an interactive care system for aged patients with dementia [16], a modular hybrid BCI based on EEG and near infra-red spectroscopy (NIRS) [17], a plug and play BCI for active and assisted living control [18], a hybrid BCI combining P300 and auditory steady state response (ASSR) [19], an embedded BCI for classification of event related synchronisation/desynchronisation [20], and a study of stimuli design for a BCI based on steady state evoked potentials (SSVEP) [21]. However, these BCIs have been tested in only a small number of participants and evaluations of the signal quality have relied on comparisons to past literature.

The breadth of research using the ADS1299 illustrates its appeal for both clinical research and end-user devices. None of these studies has evaluated the EEG recording performance of the ADS1299 in comparison with a high quality system in absence of other variables such as novel electrodes, an algorithm, or a BCI paradigm. Researchers have emphasised the need for objective evaluation of EEG signals from devices intended to be used in BCI applications [22,23,24]. Thus, there is a pressing need for an an independent evaluation of the ADS1299 with a robust experimental design. The aim of this research is to evaluate its performance in EEG against a high quality laboratory-based system while controlling for participants, paradigm, tasks performed, electrodes used, and data processing methods. This research also aims to evaluate the performance of the ADS1299 in both single joint (dorsiflexion while sitting) and multi-joint (step on/off while standing) motor tasks.

## 2. Materials and Methods

This within-participants experimental study was conducted at Auckland University of Technology, New Zealand. Ethical approval for the study (17/CEN/133) was obtained from Central Health and Disability Ethics Committee (HDEC), New Zealand, in accordance with the Declaration of Helsinki.

### 2.1. Participants

Twenty-two healthy participants (average age: 36 ± 6 years, 10 female) were recruited through professional networks and local advertising. Participants were excluded if they had a history of any neurological disorders or epilepsy. All the participants signed a written consent form before data collection.

### 2.2. EEG Systems

#### 2.2.1. Prototype

For evaluation of the ADS1299, a prototype system was built using OpenBCI (OpenBCI, New York, NY, USA) V3-32 board along with a V3 Daisy module for supporting up to 16 channels, a 4N25 optocoupler, and required connectors. The firmware of the OpenBCI board was modified from version 3.0.0 (refer to supplementary files). The data were saved on the onboard SD card. The sampling rate was set at 250 Hz and programmable gain (PGA) at 24, as these settings result in minimum peak-to-peak input referred noise [1].

#### 2.2.2. Gold Standard

NuAmps (Compumedics Neuroscan, Dresden, Germany) was used as the gold standard system (GS) [25,26,27,28,29]. A search on Google Scholar for the keywords “Compumedics Neuroscan NuAmps” returned 395 results. It was connected to a computer via a USB cable and data were recorded with the Acquire software (Compumedics Neuroscan, Dresden, Germany). As per standard data collection protocols, the sampling rate was set at 500 Hz [25,26,27,28,29].

#### 2.2.3. Electrodes

With both the systems, a 32 channel Quick-Cap with Ag/AgCl wet electrodes (Compumedics Neuroscan, Dresden, Germany) was used for EEG, and disposable BlueSensor N (Ambu^®^, Bayan Lepas, Malaysia) electrodes were used for surface electromyography (sEMG). A 37 pin D-subminiature connector was used to connect the prototype to the Quick-Cap.

### 2.3. Experiment Protocol

For each of the 22 participants, data were collected over two sessions on consecutive days. In each session, participants performed two motor tasks using a single system. The order of the systems and the order of the motor tasks was randomised across the participants. Thus, all the participants performed both the tasks using both the systems, but the order in which they performed these tasks and the choice of the system used on a given day was random. This pair-wise matching of the participants across the two systems and the two tasks allowed for the paired statistical analysis. This within-participant protocol is in agreement with the previous research evaluating EEG hardware [22,23].

In each session, the participants executed 50 right foot ballistic dorsiflexions while seated and 50 repetitions of right foot step on and off a step-stool (approximately 23 cm high) placed at a comfortable distance while standing. Participants were asked to place their right foot on the step-stool and immediately bring it back to the ground. They executed the tasks at their own pace while pausing for at least 5 s between each repetition. The order of the tasks was chosen at random, and data were recorded separately for each task.

EEG data were collected from 14 International 10–20 system locations (Fp1, F3, F4, FC3, FCz, FC4, C3, Cz, C4, CP3, CPz, CP4, P3, and P4) using either the gold standard or the prototype depending on the randomisation schedule. A single reference electrode from Quick-Cap was used, which was located on the right mastoid. Electrodes were prepared using Quick-Gel (Compumedics Neuroscan, Dresden, Germany). For sEMG, two electrodes were placed on the right Tibialis Anterior (TA) muscle. Preparation included shaving, exfoliating with the Nuprep Gel (Weaver and Company, Aurora, CO, USA), and cleansing with disposable alcohol swabs. Acquire software (Compumedics Neuroscan, Dresden, Germany) was used in combination with NuAmps to monitor impedance for both EEG and sEMG, and was accepted when below 10 kΩ. During data collection, the systems were placed on a desk next to the participant.

### 2.4. Data Processing

The data were processed on MATLAB 2017b (MathWorks, Inc., Natick, MA, USA) using a combination of custom code (refer to supplementary files) and EEGLAB (version 14.1.1) functions [30]. Movement onsets from rectified sEMG data were detected using an extended version of the double thresholding algorithm [31]. These onsets were then visually checked and adjusted. Using 2nd order, zero-phase, Butterworth filters, sEMG data were filtered with a high pass cut-off at 10 Hz, and a low pass cut-off at 200 Hz and at 100 Hz for the gold standard and the prototype, respectively. As sEMG was used only for identifying the movement onsets, these cut-offs were considered adequate [32,33].

#### EEG Processing

EEG channels were filtered with 2nd order, zero phase, Butterworth filters with a highpass filter cut-off at 0.05 Hz, a low pass filter cut-off at 40 Hz, and a notch filter from 49 to 51 Hz. These filtered EEG channels were visually inspected along with their frequency spectrum and histograms. The channels which were missing or had very large transients were removed and interpolated with EEGLAB *pop_interp* function using the *spherical* interpolation method. The data were then down-sampled to 125 Hz, and epochs were derived by including data from 3 s before and after the sEMG onset.

Then, epochs with large or very fast transients were removed and independent component analysis (ICA) was performed with EEGLAB *pop_runica* function using the *runica* algorithm [34]. Components corresponding to eye blinks, or limited to only one electrode and a few epochs, were removed. The remaining components were remixed (back-projected to sensor space), and epochs with an amplitude above 125 μV_pp_ across any channel were removed to obtain clean EEG epochs. Movement-related cortical potential (MRCP) epochs were obtained from these cleaned EEG epochs by applying a small spatial filter across FC3, FCz, FC4, C3, Cz, C4, CP3, CPz, and CP4 channels with the center at Cz [35], followed by a 2nd order, zero phase, Butterworth filter with a low pass cut-off at 5 Hz. By taking the mean across the epochs, the averaged MRCP was obtained.

### 2.5. Performance Measures

In order to compare the two systems, the following performance measures were obtained from the EEG epochs and the averaged MRCP for each participant.

#### 2.5.1. EEG Specific Measures

- Power across EEG Bands: Power across four EEG bands (delta [0.05–3 Hz], theta [3–8 Hz], alpha [8–12 Hz], and beta [12–38 Hz]) was obtained separately from each epoch for all the channels using MATLAB *bandpower* function. The obtained power was converted to decibels (dB) and then averaged across epochs and channels for each band [22].
- Pre-Movement Noise (PMN): The EEG activity from 2–3 s before the sEMG onset was regarded as baseline [36]. The root mean square (RMS) value of the baseline was calculated separately from each epoch for all the channels and then averaged across channels and epochs to obtain pre-movement noise (PMN) [37].

#### 2.5.2. MRCP Specific Measures

- Signal-to-Noise Ratio (SNR): The signal-to-noise ratio was defined as the ratio of peak negative amplitude to the RMS value in the baseline segment of the averaged MRCP, expressed in decibels.
- Amplitude and Time of Negative Peak (PN, PNT): The amplitude of the negative peak in the 1 s before and after the sEMG onset was obtained from the averaged MRCP using a local peak algorithm [38]. The time of the peak negative amplitude was expressed in milliseconds, where a negative value represents occurrence before, and a positive value after, the sEMG onset. The peak negative value is one of the most important features of the MRCP as it has been widely studied in relation with rehabilitation and motor learning [39,40].

### 2.6. Statistical Analysis

Statistical analysis was performed in R (R Foundation for Statistical Computing) version 3.5.0 (refer to supplementary files). Means are given with the standard deviations. Based on an a priori statistical analysis, the performance of the prototype was assessed against the gold standard for power across EEG bands, power ratio, pre-movement noise, signal-to-noise ratio, and the amplitude and time of the negative peak using linear mixed models or generalised linear mixed models as appropriate [41]. *lme4* package version 1.1-17 was used for fitting the models [42]. The detailed setup of the models is given in the corresponding result sections. Analysis of deviance tables for the models were obtained using the *Anova* function from *car* package version 3.0-0 [43]. Significance level was set at 0.05. For main effects and interactions, Type II Wald chi-square tests are reported. In case of significant interactions, pair-wise comparisons were performed with Tukey’s HSD (honest significant differences) method using the *lsmeans* function from the *lsmeans* package version 2.27-62 [44]. For the pair-wise comparisons, Cohen’s *d* effect size using pooled variance was calculated. It was interpreted as a small (0.20), medium (0.5), or large (0.8) effect [45]. Cosine similarity was also assessed for the averaged MRCPs across the two systems. The MRCPs from the two systems were treated as vectors and their cosine similarity was defined as follows [46].

(1) $$r=\frac{u.v}{∥u∥\times ∥v∥}$$

where **u**, **v** represent the two MRCPs as vectors. ‘**.**’ represents the dot product between the two vectors, and ∥.∥ represents the L_2_ norm of the vector. Two additional analyses which were not part of the a priori analysis plan were also performed. First, grand average MRCPs for the gold standard and the prototype were obtained in dorsiflexion and step on/off from the averaged MRCPs of the participants. These grand average MRCPs were plotted along with their differences and 95% confidence intervals. Differences were evaluated by applying sample wise repeated measure *t*-tests and interpreted based on Bonferroni corrected *p*-values. Second, interpolated topographic maps were also obtained at different latencies by task and system wise pooling of all the cleaned EEG epochs. The *pop_topoplot* function from EEGLAB was used for this purpose. Differences were evaluated by obtaining the maps from absolute difference of the channels.

## 3. Results

### 3.1. Data Loss and Artefacts

**Figure 1:**
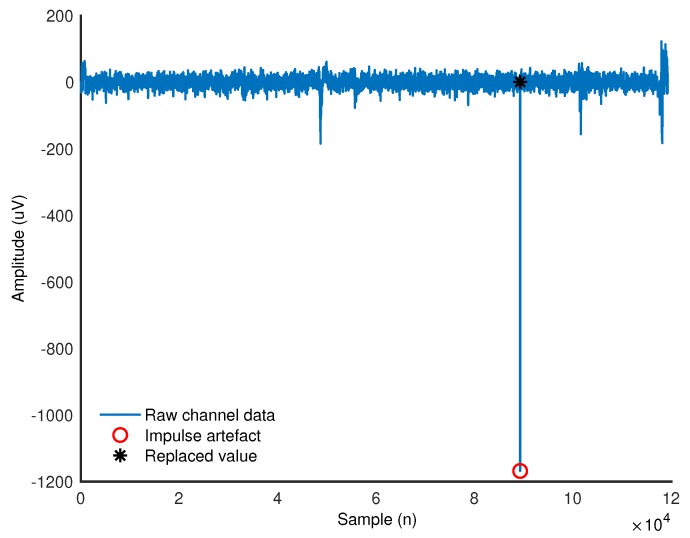
An example of the artefact detected in the prototype data.

In case of the gold standard system, there was no loss of data. For the prototype, the data from dorsiflexion of one participant were lost as the sEMG channels were recorded for only the first repetition. The EEG data from this repetition were rejected due to the presence of large transients. The prototype’s data had occasional single-sample very-large-amplitude (>1000 μV) artefacts in some channels as shown in Figure 1. The incidence of these artefacts was approximately 1 in 10,000 samples. It was removed using an automatic detection algorithm and replaced with the average of the fifth sample from its left and right.

### 3.2. Rejection of Channels, ICA Components, and Epochs

For the gold standard system, on average 0.5 ± 0.8 and 0.7 ± 1.5 channels were interpolated in dorsiflexion and step on/off respectively, across the participants. For the prototype, 0.3 ± 0.6 and 0.4 ± 0.7 channels were interpolated. During the ICA analysis, components corresponding to eye blinks, or limited to only one electrode and a few epochs, were removed. For the gold standard 3.0 ± 1.9 and 3.8 ± 2.1 components were rejected in dorsiflexion and step on/off, respectively. For the prototype, 3.4 ± 2.2 and 3.3 ± 1.8 components were removed. The number of interpolated channels and rejected components appear to be similar across systems and higher in step on/off compared to dorsiflexion.

Two sets of 50 EEG epochs corresponding to the two systems were obtained for each task from the sEMG onsets. The epoch rejection rate was calculated as the ratio of the number of epochs rejected manually and with thresholding to number of sEMG onsets used for creating epochs. This ratio was expressed as a percentage. The mean rejection rate at 125 μV_pp_ ranged from 4.9 to 7.6%. However, when 75 μV_pp_ threshold was applied, it ranged from 31.9 to 52.5% across tasks and systems, refer to Table 1. Whilst the standard deviation of the dorsiflexion rejection rate appears higher in the prototype, this can be explained by the rejection of 1 out of 1 epoch for the participant whose sEMG data were lost as explained earlier in Section 3.1.

### 3.3. EEG Specific Measures

#### 3.3.1. Power Across EEG Bands

Separate linear mixed models were set up to compare EEG power in dorsiflexion and step on/off, respectively. Systems, bands, and an interaction term for systems and bands were entered as fixed effects. For participants, a random intercept term was also entered. The setup of the linear mixed model is given in R formula syntax as follows [42].

Power ~ System + EEGBand + System ∗ EEGBand + (1|Participant)

**Figure 2:**
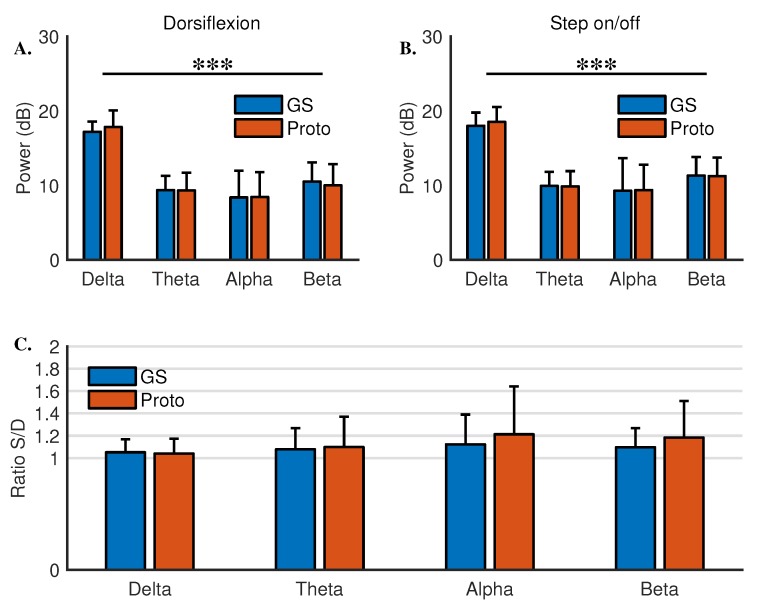
Power recorded across four EEG bands in (**A**) dorsiflexion and (**B**) step on/off. (**C**) Participant wise ratio of power values from step on/off to dorsiflexion (S/D). ‘***’ represents *p*-value less than 0.001.

In dorsiflexion, there was no interaction between the systems and EEG bands (χ^2^[3] = 1.82, *p* = 0.61). Moreover, there was no significant difference across systems (χ^2^[1] = 0.01, *p* = 0.91). There was a significant difference in power across bands (χ^2^[3] = 554.42, *p* < 0.001). Similar results were obtained in step on/off. There was no interaction between the systems and EEG bands (χ^2^[3] = 0.57, *p* = 0.90). There was no significant difference across systems (χ^2^[1] = 0.13, *p* = 0.72). There was a significant difference across the bands (χ^2^[3] = 508.33, *p* < 0.001) (see Figure 2A,B and Table 2). These results indicate that the prototype system is comparable to the gold standard system with respect to power.

In order to assess the sensitivity of the systems to detect differences in power across tasks, a participant wise ratio of power values from step on/off to dorsiflexion (S/D) was obtained, as shown in Figure 2C. For the delta band, the power ratios for the gold standard and the prototype were 1.05 ± 0.12 and 1.04 ± 0.13, respectively. The power ratios for the prototype in theta, alpha, and beta bands were 1.1 ± 0.27, 1.2 ± 0.43, and 1.18 ± 0.33, respectively. For the gold standard, the ratios for the theta, alpha, and beta bands were 1.08 ± 0.19, 1.12 ± 0.27, and 1.1 ± 0.17, respectively. A linear mixed model with systems, bands, and an interaction term for systems and bands as fixed effects was set up. A random intercept term for the participants was also entered. Significant interaction between the bands and the systems on the power ratio was not detected (χ^2^[3] = 1.51, *p* = 0.68). There were no significant differences across bands (χ^2^[3] = 6.8, *p* = 0.08) or systems (χ^2^[1] = 1.94, *p* = 0.16). These results suggest that both systems have similar sensitivity to power differences in EEG bands across different motor tasks.

#### 3.3.2. Pre-Movement Noise

Pre-movement noise in dorsiflexion was 8.82 ± 1.57 μVrms and 8.85 ± 2.00 μVrms for the gold standard and the prototype respectively. In step on/off the values for the two systems were 9.87 ± 1.91 μVrms and 10.11 ± 2.54 μVrms. To evaluate differences across systems and tasks in the pre-movement noise, a generalised linear mixed model with the Gamma family and log link was set up as the data had skew with a long tail. Systems, tasks and an interaction term for systems and tasks were entered as fixed effects. A random intercept term for the participants was also entered. The setup of the generalised linear mixed model is given in R formula syntax as follows [42].

Noise ~ System + Task + System ∗ Task + (1|Participant)

**Figure 3:**
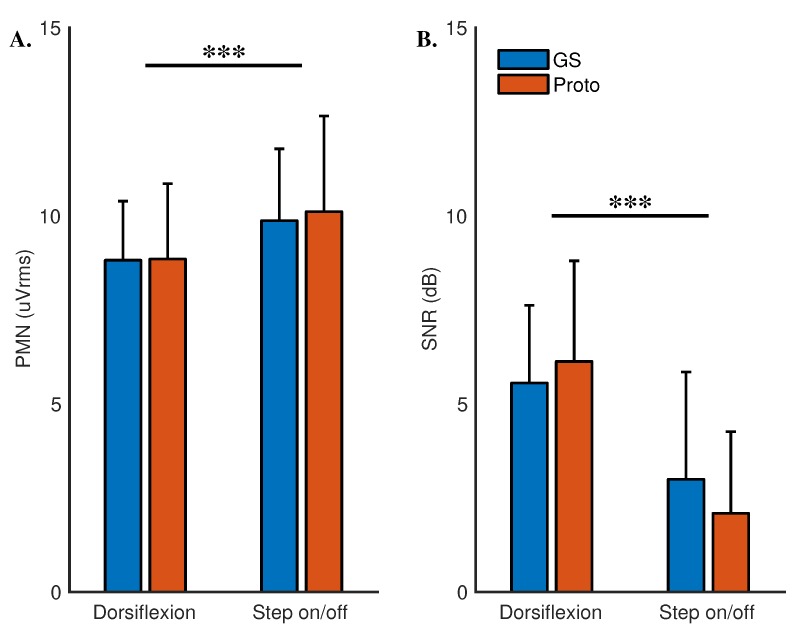
Means and standard deviations for (**A**) pre-movement noise and (**B**) signal-to-noise ratio. ‘***’ represents *p*-value less than 0.001.

There was no significant interaction between systems and tasks on pre-movement noise (χ^2^[1] = 0.09, *p* = 0.77). There was no significant difference across the systems (χ^2^[1] = 0.05, *p* = 0.83). A significant difference was detected across the tasks (χ^2^[1] = 14.12, *p* < 0.001). These results suggest that the prototype system’s susceptibility to noise is similar to the gold standard system. However, both systems have higher noise in step on/off compared to dorsiflexion, refer to Figure 3A.

### 3.4. MRCP Specific Measures

#### 3.4.1. Signal-to-Noise Ratio

Signal-to-noise ratio in dorsiflexion was 5.56 ± 2.06 dB and 6.13 ± 2.67 dB for the gold standard and the prototype respectively. In step on/off the values for the two systems were 2.99 ± 2.85 dB and 2.09 ± 2.17 dB. A linear mixed model with systems, tasks and an interaction term for systems and tasks as fixed effects was setup. A random intercept term for the participants was also entered. Significant interaction between the systems and the tasks was not detected (χ^2^[1] = 2.32, *p* = 0.13). No significant differences were detected across the systems (χ^2^[1] = 0.111, *p* = 0.74). However, a significant difference was detected across the tasks (χ^2^[1] = 44.75, *p* < 0.001). These results indicate that the signal-to-noise ratio of the prototype is comparable to that of the gold standard, refer to Figure 3B.

#### 3.4.2. Amplitude and Time of the Negative Peak

Peak negative value in dorsiflexion for the gold standard and the prototype system was −4.44 ± 2.17 μV and −4.29 ± 2.00 μV respectively. In step on/off the PN amplitude was −2.36 ± 1.34 μV and −2.75 ± 1.50 μV. A linear mixed model with systems, tasks and an interaction term for systems and tasks as fixed effects was setup. A random intercept term for the participants was also entered. Interaction between the systems and the tasks on the PN amplitude was not significant (χ^2^[1] = 1.04, *p* = 0.31). There were no significant differences across the systems (χ^2^[1] = 0.23, *p* = 0.63). A significant difference across the tasks was detected (χ^2^[1] = 47.61, *p* < 0.001).

The time of the peak negative amplitude in dorsiflexion was 261.09 ± 139.32 ms and 256.00 ± 181.73 ms for the gold standard and the prototype system respectively. In case of step on/off, the time for the systems was 284 ± 340.24 ms and 397.82 ± 344.05 ms. A linear mixed model with systems, tasks and an interaction term for systems and tasks as fixed effects was setup. A random intercept term for the participants was also entered. Interaction between the systems and the tasks on the time of the peak negative amplitude was not significant (χ^2^[1] = 1.23, *p* = 0.27). There were no significant differences across the systems (χ^2^[1] = 1.11, *p* = 0.29) and the tasks (χ^2^[1] = 2.31, *p* = 0.13).

**Figure 4:**
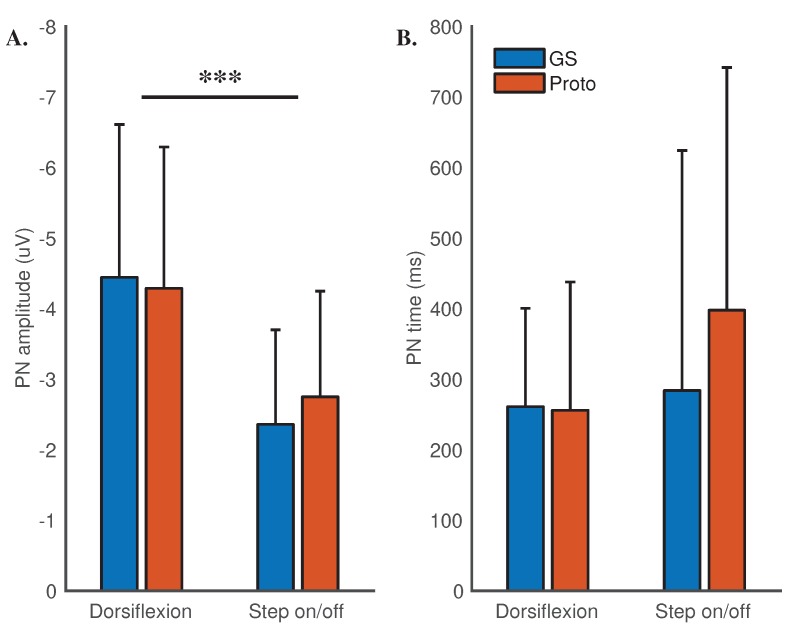
Means and standard deviations for (**A**) the peak negative value and (**B**) its time for the two systems across the tasks. ‘***’ represents *p*-value less than 0.001.

The systems were comparable with respect to the time and the amplitude of the negative peak in the MRCP. The amplitude of the negative peak differed significantly between the motor tasks, refer to Figure 4.

### 3.5. Cosine Similarity

The mean similarity in dorsiflexion across the two systems was 0.84 ± 0.14 and 0.75 ± 0.28 in step on/off. Averaged MRCPs from the two systems along with cosine similarity are given in Appendix A. The results indicate a strong cosine similarity between the MRCP signals recorded by the two systems, suggesting that the prototype is comparable to the gold standard.

### 3.6. Grand Average of Participant MRCPs

**Figure 5:**
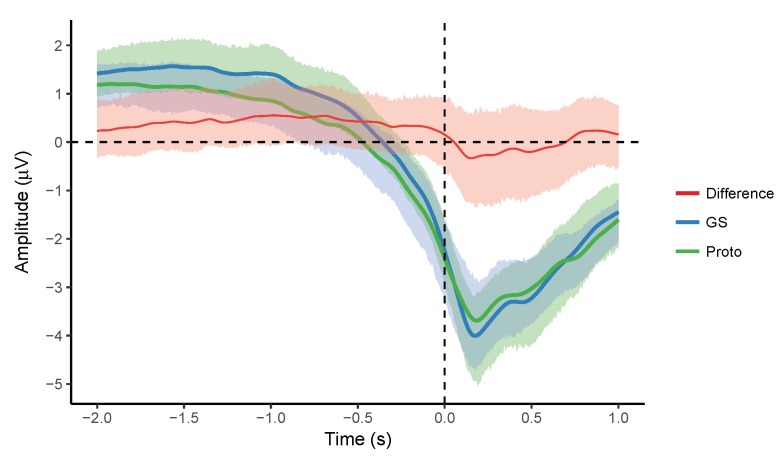
Grand averages and difference (GS-Proto) along with 95% confidence intervals for the averaged MRCPs (*n* = 21). Time at 0 s corresponds to the movement onset.

**Figure 6:**
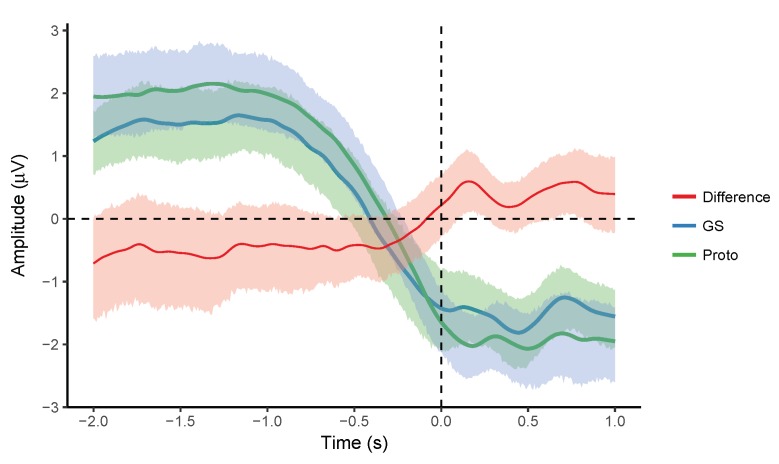
Grand averages and difference (GS-Proto) along with 95% confidence intervals for the averaged MRCPs (*n* = 22). Time at 0 s corresponds to the movement onset.

Grand averages and difference waveforms with 95% confidence intervals obtained from the averaged MRCPs of the individual participants are shown in Figure 5 and Figure 6. The grand average MRCPs were similar for the two systems in both the tasks. The confidence intervals were uniform, overlapping, and their size was smaller than ±1 μV. There were no significant differences across the systems. These results indicate agreement between the averaged MRCPs recorded by the two systems.

### 3.7. Topographic Maps

**Figure 7:**
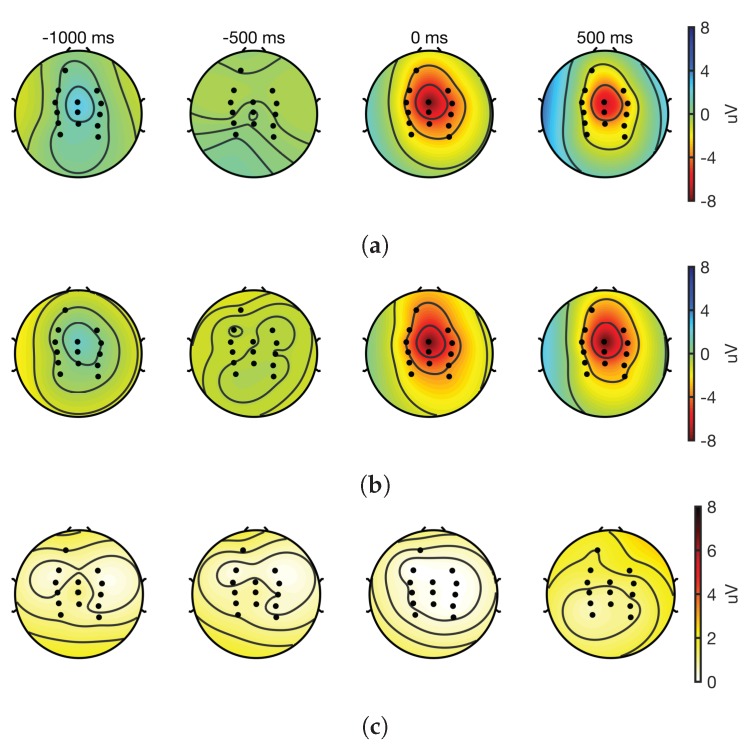
Interpolated topographic maps obtained at different latencies with respect to the movement onset form cleaned EEG epochs (*n* = 21); 977 and 1007 epochs were used for the gold standard and the prototype, respectively. Time at 0 ms corresponds to the movement onset. (**a**) GS; (**b**) Proto; (**c**) Absolute difference of channels from (**a**) and (**b**).

**Figure 8:**
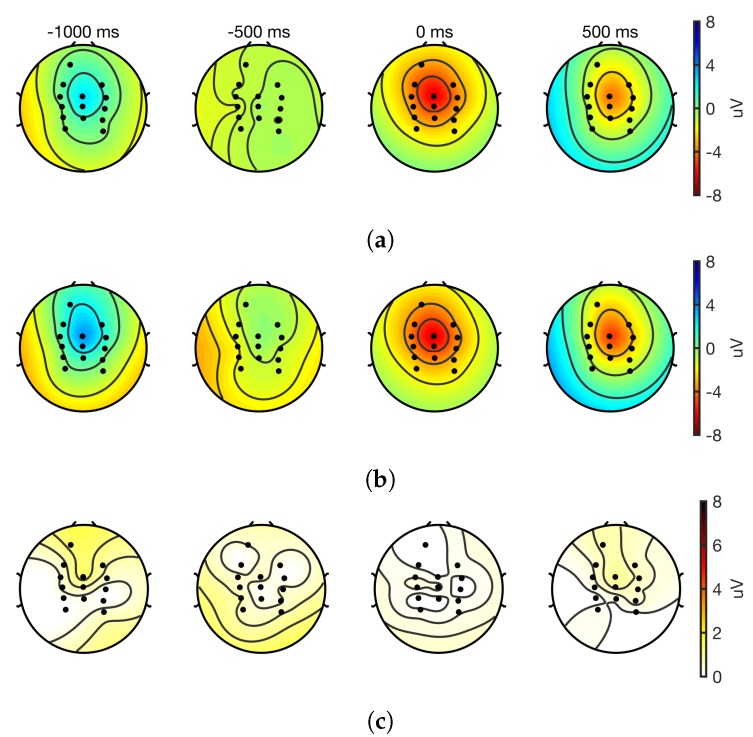
Interpolated topographic maps obtained at different latencies with respect to the movement onset form cleaned EEG epochs (*n* = 22); 1022 and 1028 epochs were used for the gold standard and the prototype, respectively. Time at 0 ms corresponds to the movement onset. (**a**) GS; (**b**) Proto; (**c**) Absolute difference of channels from (**a**) and (**b**).

Interpolated topographic maps obtained from cleaned EEG epochs of the 14 EEG channels are shown in Figure 7 and Figure 8 for dorsiflexion and step on/off, respectively. These maps suggest that in both tasks, the spatial distribution of cortical activity was similar, and the differences across the systems are negligible. At movement onset, cortical activations are centered around CPz and Cz in both systems. These results also indicate that both systems record similar EEGs.

## 4. Discussion

This experimental study has rigorously evaluated the EEG recording performance of ADS1299 in comparison to a high quality laboratory-based system whilst controlling for participants, paradigm, tasks performed, electrodes used, and data processing methods in a large sample of healthy participants. The main findings of this research are that the ADS1299 is comparable with respect to power across EEG bands, power ratio, pre-movement noise of the EEG, and the signal-to-noise ratio, the amplitude and time of the negative peak, and cosine similarity of the MRCPs. The validity of these findings are supported by the large sample size and the robust statistical analysis, which accounted for between-participant variance. These results are discussed in detail below.

We computed the average power in delta, theta, alpha, and beta bands from all the included epochs of all the recorded channels. Thus, the obtained value included both the evoked and the induced power. Within both motor tasks, there were no significant differences between the laboratory-based system and the ADS1299. The sensitivity to power changes across different tasks was evaluated with the ratio of step on/off power to dorsiflexion. There were no differences in the power ratio across the systems or the bands. Therefore, in EEG studies where spectral features during motor tasks are of interest [47,48,49], ADS1299 can be used with confidence.

Another important measure of EEG device quality is the level of noise present in the baseline. Similar to past studies [22,37], this was quantified by the root mean square value in the baseline EEG from epochs of all channels. There were no differences across the two systems. The noise was higher in the case of step on/off compared to dorsiflexion. The most likely sources of this noise are cable sway [50], head movements [51], or physiological differences. In this research, the experimental protocol and the requirement to standaradise the setup across the systems necessitated that the ADS1299 was placed on a desk. However, in mobile BCI applications, the effects of head movements and cable sway may be mitigated by the use of an inertial measurement unit and by mounting it closer to the electrodes [52]. Comparable noise levels in both systems is a strong indicator of the quality of EEG recorded by the ADS1299.

Two notable differences between the systems were revealed during data pre-processing. First, the prototype data exhibited a single sample, very large value artefact. This problem most likely originated from the way data were stored on the SD card using the OpenBCI board, as the error was also found in the raw data file before conversion from hexadecimal values. The artefact could not be removed with a low pass filter as this resulted in large transients. However, the artefact sample could easily be substituted with an averaged value. The second difference between the systems was that, in one prototype file, sEMG data were missing. The source of this problem is most likely that the Daisy board was disconnected from the main OpenBCI board and failed to reconnect during recording. In future studies, this problem can be avoided by ensuring a more robust connection between the two boards.

A comparable number of interpolated channels, rejected ICA components, and epoch rejection rates indicate that the ADS1299 records EEG reliably. We explored epoch rejection rates at traditional 75 and 125 μVpp thresholds. At 75 μVpp, both systems had rejection rates of 40–50% during step on/off, which is similar to those reported by Oliveira et al. for the Biosemi system and the Cognionics wet system during a walking task [22]. At 125 μVpp, the ADS1299 epoch rejection rate was below 10%, supporting its use in EEG devices.

MRCPs are slow moving potentials that start before the movement onset and continue during and after the movement [36]. In this research, MRCPs were recorded in motor tasks undertaken while sitting and standing. The fact that they are challenging to record and process as they are found in the delta band and are highly susceptible to movement artefacts and other sources of noise makes them an excellent test case for evaluating EEG devices. Based on the findings of this research, it can be asserted that the ADS1299 is an excellent choice for recording MRCPs. This assertion is supported by the equivalence of signal-to-noise ratio, time and amplitude of the negative peak, and the cosine similarity, the waveform differences of the MRCP with that of the laboratory-based system. Additionally, the topographic maps showed that the cortical distribution of the EEG was similar across the two systems in both tasks and concordant with previous research [26,36]. These findings have implications for the translation of MRCP-based BCI paradigms to real-world applications [39,53,54].

Reflecting on the differences between the MRCP in the two motor tasks highlighted some important findings which have implications for researchers interested in MRCP-based brain computer interfaces targeted toward both single joint movements such as dorsiflexion and functional movements such as walking [55] or sitting/standing [56]. The signal-to-noise ratio was significantly lower in step on/off, due to higher noise in baseline EEG and a smaller negative peak in the MRCP. The decrease in the peak negative value in step on/off may be explained by the torque generated in the two tasks [26], although other task parameters may have played a role as well. This is supported by the fact that the time of the negative peak was the same across both tasks, although it had larger variability in step on/off. The differences across the tasks were found in both systems, suggesting that the ADS1299 is sensitive enough to discern these variances.

The findings of this research should be considered in light of a number of limitations. First, EEG and sEMG data were not recorded simultaneously with both systems. Rather, a single system was used in a given recording session in a randomised order on different days. This protocol is in line with the research comparing different EEG devices using within-participant designs [22,23,24]. Second, the performance of an EEG recording system depends not only on the amplification chip but also on the quality of the electrode system and the design of the printed circuit board (PCB). Thus, the performance evaluation of the ADS199 performed in this research should be interpreted in relation with the used electrode system (Compumedics Neuroscan Quick-Cap) and the circuit board (OpenBCI Cyton V3-32 board). Third, the sample rate used for ADS1299 was 250 Hz and PGA gain was 24 as these settings result in minimum input referred noise [1]. On the other hand, in agreement with past research, the sampling rate was set at 500 Hz for the NuAmps [25,26,27,28,29]. To address this, data from both systems were down-sampled to 125 Hz before analysis. Fourth, the features studied in this research only represented the lower range (<40 Hz) of the EEG spectrum. Thus, further research is required to investigate the quality of EEG recordings with ADS1299 in the higher frequency bands.

## 5. Conclusions

This study has comprehensively demonstrated that the ADS1299 records low frequency (<40 Hz) EEG at a level comparable to a laboratory-based system. Using a robust experimental design with pre-planned statistical analysis, we found no significant differences across the two systems in both EEG specific measures, such as power across bands, power ratio across bands, and pre-movement noise, and MRCP specific measures, such as signal-to-noise ratio as well as time and amplitude of the negative peak. In addition, this study illustrated differences in the MRCP of the two motor tasks, one single joint, and the other multi-joint. We conclude that ADS1299 in combination with wet Ag/AgCl electrodes is a good choice for both clinical research and the development of mobile BCIs based on low frequency (<40 Hz) EEG.

## Figures and Tables

**Table 1:** Means and standard deviations for percentage epoch rejection rates at 75 μV_pp_ and 125 μV_pp_.

Task	System	Rejection at 75 μV_pp_ (%)	Rejection at 125 μV_pp_ (%)
Dorsiflexion	GS	31.9 ± 26.4	6.1 ± 7.0
	Proto	34.7 ± 31.7	7.6 ± 20.9
Step on/off	GS	52.5 ± 35.6	5.7 ± 6.2
	Proto	43.6 ± 34.1	4.9 ± 4.0

**Table 2:** Means and standard deviation for power values in decibels (dB) across four EEG bands in dorsiflexion and step on/off.

Task	System	Delta (dB)	Theta (dB)	Alpha (dB)	Beta (dB)
Dorsiflexion	GS	17.2 ± 1.4	9.4 ± 1.9	8.4 ± 3.5	10.5 ± 2.6
	Proto	17.9 ± 2.2	9.3 ± 2.4	8.5 ± 3.3	10.0 ± 2.8
Step on/off	GS	18.0 ± 1.8	9.9 ± 1.9	9.3 ± 4.4	11.3 ±2.5
	Proto	18.5 ± 2.0	9.9 ± 2.1	9.4 ± 3.4	11.3 ±2.5

**Figure A1:**
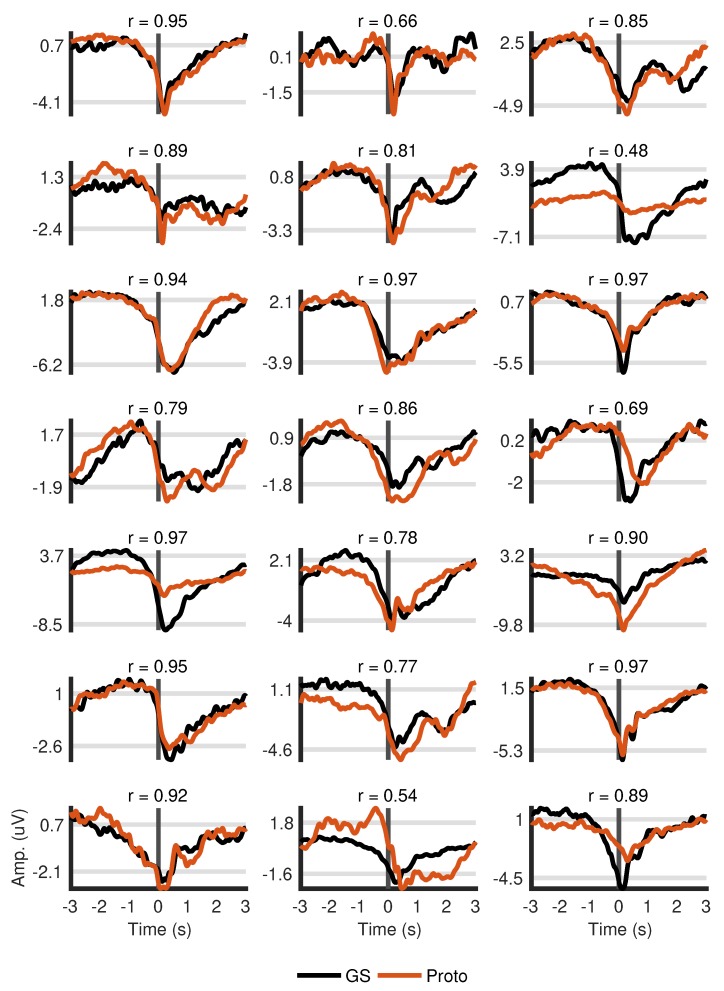
Averaged MRCPs for 21 participants in dorsiflexion from gold standard and the prototype along with cosine similarity (r). GS and Proto stand for the gold standard and the prototype system respectively.

**Figure A2:**
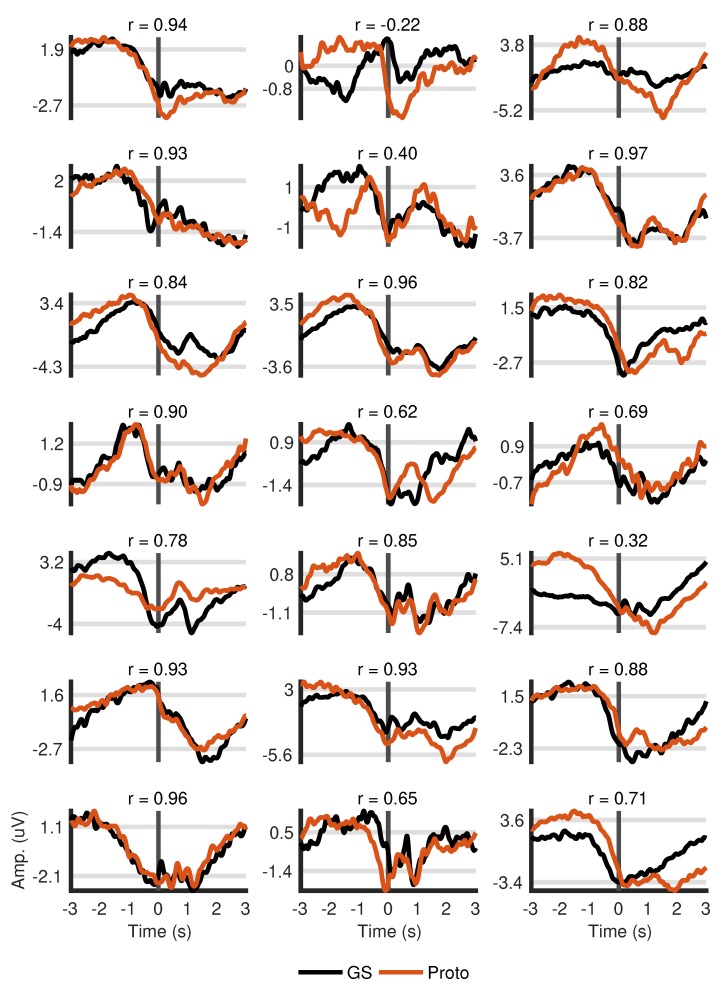
Averaged MRCPs for 21 participants in step on/off from gold standard and the prototype along with cosine similarity (r). GS and Proto stand for the gold standard and the prototype system respectively.

## Supplementary Materials

The *git* difference report between the modified firmware used for the prototype and version 3.0.0 of OpenBCI is available at http://www.mdpi.com/1424-8220/18/11/3721/s1. The data processing and statistical analysis pipeline is available on GitHub at https://github.com/GallVp/evalMRCP.

## Abbreviations

The following abbreviations are used in this manuscript:

BCI	Brain computer interface
dB	Decibels
EEG	Electroencephalography
GS	Gold standard system based on Compumedics Neuroscan NuAmps
ICA	Independent component analysis
MRCPs	Movement-related cortical potentials
ms	Milli-seconds
Proto	The prototype system based on Texas Instruments ADS1299 and OpenBCI Cyton Board V3-32
PN	Peak negative value in an MRCP
PNT	Time of PN value
PMN	Pre-movement noise measured as RMS value
RMS	Root mean square value
r	Cosine similarity
SNR	Signal-to-noise ratio
sEMG	Surface electromyography
S/D	Participant wise ratio of power values in step on/off to those in dorsiflexion
μV, uV	Micro-volts
μVrms, uVrms	Micro-volts measured as RMS value
