# Supplementary material for: An EEG Experimental Study Evaluating the Performance of Texas Instruments ADS1299
Source: Sensors (Basel). 2018 Nov 1;18(11):3721. doi: 10.3390/s18113721 (PMC6263632; doi:10.3390/s18113721)
Supplement: Supplementary file 1 [file sensors-18-03721-s001.pdf]

```
$ git diff v3.0.0
diff --git a/examples/DefaultBoard/DefaultBoard.ino b/examples/DefaultBoard/DefaultBoard.ino
index 52dd0bb..c526566 100644
--- a/examples/DefaultBoard/DefaultBoard.ino
+++ b/examples/DefaultBoard/DefaultBoard.ino
@@ -10,6 +10,7 @@
 boolean addAccelToSD = false; // On writeDataToSDcard() call adds Accel data to SD card write
 boolean addAuxToSD = false; // On writeDataToSDCard() call adds Aux data to SD card write
 boolean SDfileOpen = false; // Set true by SD_Card_Stuff.ino on successful file open
+short oldTriggerValue = 1;

 void setup() {
   // Bring up the OpenBCI Board
@@ -36,14 +37,17 @@ void loop() {
         } else {
           addAuxToSD = true;
         }
-
        // Verify the SD file is open
        if(SDfileOpen) {
          // Write to the SD card, writes aux data
          writeDataToSDcard(board.sampleCounter);
        }
-
-       board.sendChannelData();
+       //Do not send Board data; Instead send trigger when received
+       //board.sendChannelData();
+       if(board.auxData[0] != oldTriggerValue && board.auxData[0] == 0) {
+         Serial0.println("Trigger Received!!");
+       }
+       oldTriggerValue = board.auxData[0];
      }
    }
```
